# Aquatic Animal Viruses and Antiviral Immunity: A Closing Editorial

**DOI:** 10.3390/v18010127

**Published:** 2026-01-20

**Authors:** Mark P. Polinski

**Affiliations:** U.S. Department of Agriculture National Coldwater Marine Aquaculture Center, Orono, ME 04473, USA; mark.polinski1@usda.gov

## 1. Introduction

Aquatic ecosystems host the planet’s greatest animal diversity, and with it, a remarkably varied virosphere. Nevertheless, across commercial and conservation aquaculture, decisionmakers are faced with three common persistent challenges: (i) distinguishing viral discovery from disease relevance, (ii) quantifying and acting on immunity at a farm/population scale, and (iii) integrating host genetics, environment, and biosecurity into coherent control strategies. This Special Issue was launched to address these challenges by advancing our knowledge of aquatic animal–virus interactions and the antiviral defenses that shape disease outcomes across fish, crustaceans, and mollusks. The assembled papers (11 in total) collectively highlight advances in virus discovery, mechanistic pathogenesis, immune monitoring, and interventions that are directly applicable to aquaculture health management, pathogen surveillance, and environmental conservation.

## 2. Motivations for This Special Issue

Metagenomics is redefining the diversity and evolution of fish viruses, highlighting long host-specific divergence patterns that juxtapose with cross-species transmission at the domestic–wild interfaces of aquatic systems [1]. These insights underscore how emergence is driven not only by viral genetics but also by aquaculture practices and environmental change. In parallel, molecular methods—from classical PCR to field-portable LAMP and next-gen sequencing —have matured, enabling more rapid identification and increased specificity for viral detection within aquatic hosts [2]. Progress in fish vaccinology (including recombinant and nucleic-acid platforms) is notable, although challenges remain for mucosal delivery, durability under stress, and implementation in the diverse species makeup of conservation and commercial aquaculture [3,4]. Here, comparative immunology continues to clarify innate and adaptive responses in fish and shellfish, laying foundations for targeted immunomodulation and selective breeding [5,6].

Yet, despite these advances, fundamental gaps persist. One challenge is that viral detection does not equal disease—many newly identified viruses lack clear pathogenic attribution, leaving managers unsure how to prioritize monitoring and mitigation [7,8]. A second challenge is that immunity is hard to measure at the population scale—laboratories may deliver precise assays, but farms require cost-effective, rapid immunometrics that inform real-time decision making within their budgetary capacity [9]. Third, host genetics matter. Resistance and susceptibility can vary dramatically by stock or family [10], yet validated workflows to incorporate genotype information into management—particularly in conservation aquaculture—are limited. Fourth, biosafety at conservation hatcheries is often challenged by latent infections and stress-modulated shedding, where policies and budgetary capacities are often insufficient to fully mitigate long-term reservoir risks [11]. Finally, translational therapeutics (applying laboratory discovery to practical application) beyond vaccines are under-tested in aquaculture, even when cell-level mechanisms suggest tractable antiviral targets.

## 3. Special Issue Synthesis

### 3.1. From Discovery to Surveillance and Pathogenesis Model Development

Two papers in this issue extend the U.S. marine virome and directly inform surveillance priorities. Lovy et al. [12] detected the Betanodavirus sequence and characterized a novel Nervous necrosis virus (NNV) genotype—provisionally BSBNNV—in black sea bass along the mid-Atlantic coast of North America, emphasizing the need to evaluate epidemiology and virulence in a region with expanding marine aquaculture. Raines et al. [13] identified three novel viruses from recently described nackednavirus and adomavirus lineages in a hyperpigmented largemouth bass lesion, advancing the concept that cryptogenic viruses are present “in plain sight” and should be folded into risk assessments for centrarchids. This has been further supported by independent identification of a similar nackednavirus in African cichlids this same year [14]. A third paper, by He et al. [15], moved from discovery and genomic/phenotypic characterization of multiple novel Dutch Cyprinid herpesvirus (CyHV-2) strains to establish an immersion-challenge model with consistent generation of ~40% goldfish mortality—allowing future vaccine and therapeutic evaluation tests to be conducted under conditions emulating natural transmission.

### 3.2. Actionable Mechanisms for Antiviral Development

Zhang et al. [16] demonstrated that an Iridovirus, ISKNV, triggers ferroptosis by suppressing GPx4 and promoting ACSL4 in CPB cells, and that pharmacological inhibition of this pathway suppresses viral yield. This finding positions ferroptosis modulation as a promising host-directed antiviral strategy in aquaculture species where vaccine solutions are incomplete or slow to deploy. Niu et al. [17] showed that the mandarin fish scTRIM44 gene positively regulated rabdoviral (SCRV) transcription despite also positively regulating RNA-virus detection pathways (RIG-I/MDA5-mediated interferon signaling). Interestingly, scTRIM44 proved inconsequential to two DNA iridoviruses (ISKNV and LMBV), revealing virus-specific host pathway effects that could be exploited for resistance or immunomodulation depending on the type of infecting virus (dsDNA vs. ssRNA).

### 3.3. Immunometrics and Rapid-Response Biologics for Improved Aquaculture

Yamkasem et al. [18] translated lab immunology into farm-level immune monitoring by validating a pooled-serum ELISA antibodies for the impactful Tilapia Lake Virus (TiLV). The work provides clear guidance on pooling sensitivity and cost-efficiency—an operational template that can be employed for other pathogen screening programs that could encompass individual to population-scale testing. Setthawong et al. [19] improved TiLV mitigation approaches by developing specific IgY antibodies extracted from laying hen egg-yolks targeting segment-4 of the virus. These antibodies demonstrated dose dependent neutralization and cytopathic effect inhibition in vitro for TiLV, providing a putative outbreak response tool that could serve as a bridge or adjunct to vaccination, particularly where licensing or delivery of vaccines is constrained.

### 3.4. Host Genetics as a Determinant of Disease

Polinski et al. [6] provided experimental evidence that Atlantic salmon stock strongly influences PRV-1/HSMI severity, independent of viral subtype (PRV-1a or PRV-1b), underscoring the value of selective breeding and stock choice for reducing disease burden. Complementing this, Toubanaki et al. [20] probed NNV re-infected transcriptomes in European sea bass, identifying distinct gene networks in resistant vs. susceptible families for use in developing biomarkers and breeding targets that promote betanodavirus resistance.

### 3.5. Biosecurity—Recognizing Acute Reservoirs and Assessing Economic Costs

Shavalier et al. [21] documented the ~8.5-year persistence of a herpesvirus (SalHV-3) in lake trout that survived an initial disease (EED) outbreak, with stress-linked intermittent detection across fins/mucus, nervous tissues, and water. This provided evidence that survivors can act as long-term reservoirs capable of shedding under routine hatchery handling, and invites re-examination of quarantine, screening periodization, and release criteria in conservation aquaculture programs. Rahaman et al. [22] also re-examined biosecurity practices at a national scale by quantifying the economic burden of viral diseases in Australian fish and prawn aquaculture and critically assessed surveillance, diagnostics, and exclusion strategies—arguing for genetics-based resistance, modern vaccines, and integrated risk frameworks that harmonize policy with farm realities. The conclusions are transferrable to other national contexts facing similar scale-up pressures.

## 4. Closing

The studies of this Special Issue showcase descriptive knowledge to aid management decisions by adding realistic challenge models, identifying mechanistic antiviral levers, demonstrating actionable immunometrics, validating host genetic effects, exposing latent reservoirs, and connecting disease biology to national biosecurity planning. They show that practical impact comes from linking discovery to surveillance, aligning mechanisms with management, and embedding genetics and ecology in day-to-day decisions. These studies also highlight the continued need for forward progress to standardize immunosurveillance, develop host-directed antivirals, integrate genetic resilience, and safeguard conservation programs so that aquaculture and stewardship can better anticipate viral change rather than react to it.

## Data Availability

No new data were created or analyzed in this study. Data sharing is not applicable to this article.
